# Quality of and barriers to routine childbirth care signal functions in primary level facilities of Tigray, Northern Ethiopia: Mixed method study

**DOI:** 10.1371/journal.pone.0234318

**Published:** 2020-06-12

**Authors:** Haftom Gebrehiwot Weldearegay, Alemayehu Bayray Kahsay, Araya Abrha Medhanyie, Hagos Godefay, Pammla Petrucka

**Affiliations:** 1 Mekelle University, College of Health Sciences, Mekelle, Ethiopia; 2 Tigray Region Health Bureau, Tigray, Ethiopia; 3 University of Saskatchewan, College of Nursing, Canada and Adjunct Nelson Mandela African Institute of Science and Technology, Tanzania, Canada; University of Oxford, UNITED KINGDOM

## Abstract

**Background:**

Efforts to expand access to institutional delivery alone without quality of care do not guarantee better survival. However, little evidence documents the quality of childbirth care in Ethiopia, which limits our ability to improve quality. Therefore, this study assessed the quality of and barriers to routine childbirth care signal functions during intra-partum and immediate postpartum period.

**Methods:**

A sequential explanatory mixed method study was conducted among 225 skilled birth attendants who attended 876 recently delivered women in primary level facilities. A multi stage sampling procedure was used for the quantitative phase whilst purposive sampling was used for the qualitative phase. The quantitative survey recruitment occurred in July to August 2018 and in April 2019 for the qualitative key informant interview and Focus Group Discussions (FGD). A validated quantitative tool from a previous validated measurement study was used to collect quantitative data, whereas an interview guide, informed by the literature and quantitative findings, was used to collect the qualitative data. Principal component analysis and a series of univariate and multivariate linear regression analysis were used to analyze the quantitative data. For the qualitative data, verbatim review of the data was iteratively followed by content analysis and triangulation with the quantitative results.

**Results:**

This study showed that one out of five (20.7%, n = 181) mothers received high quality of care in primary level facilities. Primary hospitals (β = 1.27, 95% CI:0.80,1.84, p = 0.001), facilities which had staff rotation policies (β = 2.19, 95% CI:0.01,4.31, p = 0.019), maternal involvement in care decisions (β = 0.92, 95% CI:0.38,1.47, p = 0.001), facilities with maternal and newborn health quality improvement initiatives (β = 1.58, 95% CI:0.26, 3.43, p = 0.001), compassionate respectful maternity care training (β = 0.08, 95% CI: 0.07,0.88, p = 0.021), client flow for delivery (β = 0.19, 95% CI:-0.34, -0.04, p = 0.012), mentorship (β = 0.02, 95% CI:0.01, 0.78, p = 0.049), and providers’ satisfaction (β = 0.16, 95% CI:0.03, 0.29, p = 0.013) were predictors of quality of care. This is complemented by qualitative research findings that poor quality of care during delivery and immediate postpartum related to: work related burnout, gap between providers’ skill and knowledge, lack of enabling working environment, poor motivation scheme and issues related to retention, poor providers caring behavior, unable translate training into practice, mismatch between number of provider and facility client flow for delivery, and in availability of essential medicine and supplies.

**Conclusions:**

There is poor quality of childbirth care in primary level facilities of Tigray. Primary hospitals, facilities with staff rotation, maternal and newborn health quality improvement initiatives, maternal involvement in care decisions, training on compassionate respectful maternity care, mentorship, and high provider satisfaction were found to have significantly increased quality of care. However, client flow for delivery service is negatively associated with quality of care. Efforts must be made to improve the quality of care through catchment-based mentorship to increase providers’ level of adherence to good practices and standards. More attention and thoughtful strategies are required to minimize providers’ work-related burnout.

## Introduction

The period around childbirth and the first 24 hours postpartum remains a perilous time for both mother and newborn despite global efforts and improvements in mortality over the past two decades [1–2]. Previously, adverse outcomes were thought to result primarily from delivery occurring outside of health facilities and from lack of access to skilled care [3–4]. However, a recent literature review showed more women in low income countries are delivering in facilities but this shift has not been consistently linked to mortality reduction nor guaranteed that appropriate interventions are rendered during the intra-partum and immediate postpartum period in settings which may result in low quality of care (QoC) [5–7]. Strategies such as the Janani Suraksha Yojana (JSY) program, a large conditional cash transfer programs in India, have increased rates of facility-based childbirth without significantly decreasing maternal and neonatal mortality [6, 8]. This finding implies improving the QoC provided during facility-based childbirth is key to decreasing maternal and neonatal mortality and complications [9,10].

Measuring QoC for mothers and newborns is multi-dimensional and conceptually complex, reflecting both the provision (in terms of structure, process and outcomes) and experiences of care. According to Donabedian, the measurement of process (how care is delivered) is nearly equivalent to the measurement of QoC because process encompasses all acts of health care delivery [11,12]. Furthermore, one approach to simplify this complexity is to focus on the content of care received as defined by the routine processes that are recommended to occur during a contact between health care user and provider. It has been argued that these processes provide a measurable and meaningful indication of QoC since they describe the potential for health-gain in given clinical scenario. In the context of maternal and newborn health care it is possible to define content of care by drawing on the global recommendations for minimum packages of routine care that should be made available to all women and newborns during intra-partum and immediate postpartum care [13,14].

The use of evidenced-based practices for routine care and management of complications is the key to achievement of high QoC [15]. Receiving a QoC is also a universal human right and should apply to all women everywhere. All women and newborn have a right to access a QoC that enables a positive childbirth experience that includes respect and dignity, a companion of choice, clear communication with maternity staff, pain relief strategies, mobility in labor, and position of choice [16,17]. Recent evidence indicated that poor quality of care at primary health care facilities, the first point of contact with the health system, not only jeopardizes the health of mothers and newborns, but erodes trust, resulting in bypassing health facilities and potentially puts the entire healthcare system and population at risk [18].

Despite mothers attending facilities for deliveries, there is mismatch between maternal and newborn services provided and demand for QoC due to limited skilled providers, knowledge deficits, lack of confidence, environment lacking consistent structured education at pre-service and in service, and lack of access to evidence based current information [6, 19]. In addition, motivation of health workers to translate knowledge into action is an important influencer of quality of care [20,21]. In Ethiopia a great deal of attention and investment of resources has been directed towards basic emergency obstetrics care training. However, this initiative alone has not translated into improved QoC. This discrepancy has given rise to the quality gap in maternal and newborn care implying that the content of care provided in health facilities is often of insufficient quality to have a major impact on avoidable deaths and complications. Therefore, in recent years a more focused attention to QoC has been raised [6, 22]. Few studies have been conducted on QoC of routine childbirth care functions and almost all of them did not use a validated instrument for measuring the QoC, making comparisons difficult [23]. Consequently, identifying the possible improvement pathways of the QoC during this critical period could have a substantial impact on maternal and newborn survival and inform hospital managers, professionals, researchers and policy makers about where improvements may be focused to enhance effectiveness of health services. Therefore, assessing the QoC provided routinely for uncomplicated childbirth and identifying barriers to QoC in primary health facilities of Tigray, Northern Ethiopia is essential.

## Materials and methods

A facility based cross-sectional explanatory sequential mixed method study was conducted among recently delivered mothers in primary health care facilities of Tigray regional state, Northern Ethiopia. Tigray is the northern most region of Ethiopia with an estimated total population of 5,247,005 with 21.2% of the population living in urban areas and 50.3% being female [24]. The maternal and new born health care services in the region are provided mainly by emergency obstetric surgeons and obstetricians at hospitals and health centers by midwives, nurses, and health officers. The service is given free of charge in all public health facilities on a seven day per week 24 hours a day basis. As of 2016, in Tigray Region, there were 2 comprehensive specialized hospitals, 15 general hospitals, 23 primary hospitals, 214 health centers, and 718 health posts [25, 26].

### Quantitative phase

a sample size calculation for the quantitative study component among recently delivered mothers was determined by a single population proportion with 95% confidence interval, margin of error (d) of 5% and taking 54.06% prevalence (P) of overall quality of delivery care in Arbaminch, south Ethiopia public health facilities [27]; design effect of 2 and adding 10% for non-response rate. A total of 881 mothers received routine intra-partum and immediate postpartum care signal functions from 40 primary level care facilities. Additionally, a total of 225 skilled birth attendants (SBAs) working in the study facilities at the time of data collection were included.

A multi-stage sampling procedure was adopted to select the districts and primary level health facilities from each district. In the first stage, three of the seven zones were selected randomly. In the second stage, nine of the 22 districts were chosen and 6 primary hospitals were randomly selected. Thereafter, all health centers with their respective catchment primary level hospitals were included with the total sample size being distributed over each of the health facilities proportionate to their sample considering average number of deliveries per facility per month. All SBAs in the study were enrolled. Finally, all eligible recently delivered women were chosen by a systematic random method until the required sample size was achieved. A referred mother requiring care at a higher level facility for further management and/or delivered by cesarean section were excluded from this study. Client exit interview tracer indicators for routine childbirth care signal functions (care that should be provided for all mothers and newborns) utilized self-administered questionnaires, facility inventories, and interviews of providers to collect the quantitative data. A 40-item knowledge tests, as well as satisfaction of health workers and facility readiness surveys (i.e., availability of infrastructure; essential medications and commodities; guidelines; staff) were conducted. Tracer indicators for facility readiness were used from the WHO Service Availability and Readiness Assessment (SARA) list, previously reported indices [28]. Twelve data collectors and three supervisors worked as data collection teams. Data collectors had previous research experience and trained for two days. Data for this quantitative study was collected between July to August 2018.

### Qualitative phase

We developed semi-structured questionnaires to conduct key informant interviews (KII) and Focus Group Discussions (FGD). Participants for FGDs and KIIs were selected purposively. The key informant participants were medical directors from each of the primary level health facilities, woreda, and regional health bureau maternal and child health experts and unit head of maternity wards. We assumed that these KII could better inform us of barriers to provide childbirth care than other health workers. A total of twelve KIs were conducted. Probing questions were used for a better understanding where necessary. Each participant was interviewed individually at his/her place of work, with interview duration ranging from 20 to 35 minutes by a team of trained data collectors. After training, three researchers from the College of Health Sciences at Mekelle University and Tigray Health Research Institute conducted qualitative data collection from the 9th to 29^th^ of April 2019. The semi-structured interview guide used can be found in **S1 Appendix.**

After the interviews, three FGD were held for SBAs working at intra-partum and immediate postpartum care ranging from 60 to90 minutes. One interviewer and note taker were involved. Skilled birth attendants with clinical work experience of six months and below were excluded from the qualitative study. All interviews and discussions were audio recorded, then transcribed verbatim in Tigrigna (the local dialect) by two independent investigators. A third investigator checked the consistency of the transcripts and verified the transcripts by listening to the tapes again. They were subsequently translated into English prior to analysis.

### Variables and measurements

The primary outcome investigated was quality of routine childbirth and immediate postpartum care. It was measured as a continuous variable constructed as a composite variable from the total of 32 standards of quality process of care indicators. The routine intra-partum and immediate postpartum care signal functions used in this study are grounded in validated indicators in the Tigray regional state context. Detail of the measurement and validated tool findings is found in the recent article submitted for publication [29]. Principal component analysis (PCA), the most common technique of creating a single or composite quality index, which is a variable reduction method to obtain a smaller set of uncorrelated variables from a large list of correlated variables, was used. Each component is a linear combination of the observed variables optimally weighted to account for the maximum amount of variance [30]. Therefore, quality measures reflect the minimum standards of routine intra-partum and immediate postpartum care, irrespective of the type of health facilities where the delivery service is performed. According to the PCA, QoC was defined as a binary variable of “low” to “high” on a continuous scale from 0 to 100. If a mother’s review received 75% and above, it was termed as high QoC, and otherwise received low QoC. Details of the PCA tool for measuring QoC is found in **S2 Appendix.**

The providers' satisfaction variable was classified as “satisfied” (providers scored 75^th^ percentile and above), whereas below the 75th percentile was considered “not satisfied”; facility readiness was categorized as adequately ready at the 75^th^ percentile and above and below was considered inadequately ready). Details of the PCA tool for measuring providers’ satisfaction is found in the **S3 Appendix.**

Knowledge of providers on intra-partum and immediate postpartum care signal functions was determined using a set of 31 multiple choice questions and 9 true or false questions. Each correct answer was valued at one point, and a wrong answer attracted no points. Questions that were not answered were treated as wrong answers. Ultimately, participants were evaluated out of 100, and grouped as either sufficient knowledge (median or higher) or insufficient knowledge (less than median value).

### Data management and analysis

#### Quantitative analysis

First, we entered the data in to EPI data, cleaned and analyzed it using SPSS™ version 21 software. Descriptive statistics were used to summarize the characteristics of delivered mothers, facilities, and providers. Characteristics of the study population were presented with mean and standard deviation for variables with normal distribution. The normality of distribution of quantitative variables was tested by Kolmogorov–Smirnov test. We used linear regression analysis to assess the association between quality of care and explanatory variables. Simple linear regression analyses were conducted and those independent variables with p value of ≤ 0.25 were considered for multiple linear regression with the forward likelihood ratio method. Finally, statistical significance was considered if p < 0.05.

Furthermore, an index score of PCA was done after checking the suitability of the data. The correlation coefficient was set at a cut-off point of 0.4 or above. The Kaiser-Meyer-Oklin value, which was used to assess sampling adequacy, was set at a cut-off point of 0.5 [30], while the Bartlett’s test of sphericity was used to support the factorability of the correlation matrix. Furthermore, a scree plot tests and eigenvalue of over 1.0, which represents the total variance explained by a factor, were used to inspect the plotting of each eigenvalue of the factors to find a point at which the shape of the curve changes direction and becomes horizontal. All factors above the break in the plot and/or with eigenvalues over 1.0 were retained for further analysis. Lastly, further analysis was done using the Vari-max method to minimize the number of variables with high loadings on each factor.

### Qualitative analysis

Two researchers independently reviewed the audio recorded comments line- by- line and then agreed on a set of codes; broadly categorized into those related to the quantitative checklist and codes for other emerging issues. Both researchers then jointly coded all the open-ended comments. In cases where disagreements arose between researchers, further discussion took place until consensus was achieved. The data analysis was carried out in three stages. First, familiarization involving reading and re-reading the transcripts to aid understanding of the data. Second, organizing and coding the data. The coding was determined based on the quantitative results, to aid understanding how the quantitative findings were manifest. The coding was done using Atlas ti™7.5 software. Third, data from each code point were reviewed and summarized to reduce the number of words without losing the content or context of the text and to ensure contents were internally consistent. Then content analysis and triangulation of data were done through a continuous back and forth interpretation of findings.

### Ethics approval and consent

The study protocol was approved by the Institutional Research Review Board of Mekelle University’s College of Health Sciences and Community Services Ethical Review Committee (ERC 1436/2018). Permission was obtained from all relevant authorities in the Tigray Regional Health Bureau and health facilities. Informed consent was obtained from all participants prior to enrollment in the study. Parental or legal guardian consent was obtained for participants who were under 18 years of age. Data collection was conducted confidentially while data was de-identified and de-linked with storage in a secure location.

## Results

### Socio-demographic characteristics of mothers

A total of 876 mothers who delivered in the primary health care facilities were included in the study with a response rate of 99.43%. Above half of the mothers (n = 465, 53.1%) were within the age group 25–34 years and ranged from 17 to 45 years (mean age = 28.9, SD = 6.1). Two thirds of the mothers (587; 67.0%) lived in rural setting and 610 (69.6%) were housewives. More than three-quarters (n = 789, 89.5%) of the participants were married, 91% (n = 798) belonged to the Orthodox Christian religion, and nearly 40% (n = 343) had no formal education. More than six out of ten mothers 566 (64.6%) walked greater than 30 minutes to the nearest health facility **[Table 1].**

**Table 1:** Socio-demographic characteristics of mothers in Northern Ethiopia, 2019 (N = 876).

Variable	Number	Percentage
**Age in years**		
15–24	227	25.9
25–34	465	53.1
35 and above	184	21.0
**Residence**		
Rural	587	67.0
Urban	289	33.0
**Mother's occupation**		
Housewife	610	69.6
Employed	158	18.0
Daily worker	108	12.4
**Marital status**		
Married	784	89.5
Single/never married	43	4.9
Divorced/widowed/separated	49	5.6
**Religion**		
Orthodox Christian	798	91.1
Muslim	59	6.7
Others*	19	2.2
**Mother's education**		
No formal education	343	39.2
Elementary school	282	32.2
Secondary school and above	251	28.6
**Estimated walking time to the nearest health facility**		
30 minute and below	310	35.4
Greater than 30 minutes	566	64.6

*Other religions = Catholic and Protestants

### Reproductive history of mothers

Seven hundred ninety-seven (91.0%) mothers had antenatal care (ANC) visits for their current pregnancy with 30.6% (n = 242) having four or more ANC visits. Seven out of ten mothers (n = 561) were gave birth in the same facility where they received ANC follow up. Around two thirds (n = 535) of women had birth preparedness and complication readiness plan. Over one third (n = 379) of the participants had between two and four pregnancies. While 510 (58.2%) mothers had between 2 to 5 children ever born. Around two out of ten (161; 18.6%) mothers had a history of abortion, while 11.4% (n = 100) had a history of stillbirth. With respect to allowing partners to enter the delivery room, about two thirds (n = 594) of women had allowed their partners to enter and receive support in the delivery room. Around three fourths of mothers (n = 664) had been involved in decision making for the type of care they received during childbirth and soon after. With respect to obstetrical complications, one hundred seven (12.2%) of mothers had faced an obstetrics complication. Of those, pregnancy induced hypertension was the most common obstetrical complication (n = 38, 35.5%) [**Table 2].**

**Table 2:** Reproductive history of mothers in Northern Ethiopia, 2019 (n = 876).

Variables	Number	Percentage
ANC visit for the current pregnancy		
Yes	797	91.0
No	79	9.0
Number of ANC visits		
1	331	41.9
2–3	217	27.5
4 and above	242	30.6
Place where ANC was received		
Health Center	487	61.1
Hospital	283	35.5
Health Post	27	3.4
Does your last ANC visit was in this facility?		
Yes	561	70.4
No	236	29.6
Birth preparedness and complication readiness (BPCR)		
Yes	535	61.1
No	341	38.9
Length of labor		
<12 hours	758	86.5
≥12 hours	118	13.5
Mode of Delivery		
Spontaneous vaginal delivery (SVD)	760	86.8
Instrument delivery	116	13.2
How long do women generally stay at the facility following a normal delivery?		
<6 hours	508	58.0
6–24 hours	282	32.2
>24 hours and above	86	9.8
Number of pregnancies/Gravidity		
1 Pregnancy	113	20.1
2–4 Pregnancies	379	67.6
5 and above pregnancies	69	12.3
Number of deliveries/Parity		
Primipara (1 delivery)	230	26.3
Multipara (2-5deliveries)	510	58.2
Grand multipara (5 and above deliveries)	136	15.5
History of abortion		
Yes	163	18.6
No	713	81.4
History of stillbirth		
Yes	100	11.4
No	776	88.6
Mothers allowed their partner to enter to the delivery room		
Yes	594	67.8
No	282	32.2
Maternal involvement in care decisions		
Yes	664	75.8
No	212	24.2
Complication(s) during the current pregnancy		
Yes	107	12.2
No	769	87.8
Type of complication(s) (n = 107)		
Hemorrhage	24	22.4
Pregnancy Induced Hypertension	38	35.5
Infection	24	22.4
Others*	21	19.6

Others complication*: Anemia, tear, delay of expulsion of placenta and head ache

### Socio-demographic characteristics of skilled birth attendants

The average age of SBAs was 29.7 years (SD ± 7.0) with a range of 21 to 58 years. The majority of SBAs (55.1%) were between 25 and 35 years. Health providers at delivery were predominantly staff midwives (52.4%).

Over half of SBAs (51.6%) providing intra-partum care were registered diploma holders and around two thirds (n = 148) of the providers attended regular program education.

Most of the SBAs (52.0%) had worked in the obstetrics unit providing intra-partum and immediate postpartum care for 2 to 5 years **[Table 3].**

**Table 3:** SBAs’ background characteristics working at obstetrics in primary health facilities of Tigray, Northern Ethiopia, 2019 (N = 225).

Variable	Number	Percentage
Age of provider in completed years		
≤ 25	62	27.6
25–35	124	55.1
> 35	39	17.3
Marital status		
Married	119	52.9
Divorced	15	6.7
Single	91	40.4
Provider work experience in years		
Less than 5 years	117	52.0
5 years and above	108	48.0
Sex of provider		
Male	75	33.3
Female	150	66.7
Highest level of education		
Diploma	116	51.6
Degree and above	109	48.4
Educational program attended		
Generic	148	65.8
Upgrade regular	48	21.3
Upgrade in-service	29	12.9
Professional cadre		
Midwife	118	52.4
Nurse	69	30.7
Health officer and MD	38	16.9

### Barriers of skilled birth attendants to QoC

Table 4 shows that about three fourths of the SBAs [74.7% (n = 168)] were dissatisfied with their existing job. Six out of ten of the SBAs (n = 137) reported ever attending a formal basic emergency obstetrics and newborn care training, followed by neonatal resuscitation or helping babies breathe (42.7%) during the past two years. One hundred and four (46.2%) of providers were knowledgeable on basic obstetrics care practices. The average SBAs knowledge score on routine intra-partum and immediate postpartum care functions was 22.61(±5.4) with the range scored from 9 to 37 out of a total 40 item questions. **S4 Appendix** shows the basic emergency obstetrics standard questionnaire/tool used for assessing the knowledge of SBAs. The qualitative result revealed that perceived lack of legal protection in terms of medical indemnity insurance, poor motivation, or benefit packages (risk allowance, low salary and lack of opportunity for further education etc..), lack of an enabling environment, poor leadership and governance, lack of capacity building mechanisms, and mismatch of number of providers and facility capacities to conduct deliveries were the main barriers of satisfaction of providers.

**Table 4:** Readiness of SBAs working in obstetrics at primary health facilities of Tigray, Northern Ethiopia, (N = 225).

Variable	Number	Percentage
In the last 2 years received basic emergency obstetrics training		
Yes	137	60.9
No	88	39.1
Neonatal resuscitation/helping babies to breathe training		
Yes	96	42.7
No	129	57.3
Compassionate respectful care training		
Yes	80	35.6
No	145	64.4
Quality improvement training		
Yes	42	18.7
No	183	81.3
Legal issues fear to make decision in your daily basis		
Yes	50	22.2
No	175	77.8
In the last 6 months, received clinical mentorship		
Yes	108	48.0
No	117	52.0
Support supervision in the last 6 months		
Yes	154	68.4
No	71	31.6
Had regular case presentation in your team/facility		
Yes	97	43.1
No	128	56.9
Recommendation to improve quality of obstetrics care		
Pre service/in-service training	89	39.6
Catchment based mentorship	115	51.1
Supportive supervision	21	9.3
Having challenge in providing intra-partum, and immediate postpartum care?		
Yes	79	35.1
No	146	64.9
Postnatal women checked and discharged by senior staff of the facility		
Yes	70	31.1
No	155	68.9
Satisfaction of skilled birth attendants		
Satisfied	57	25.3
Not satisfied	168	74.7
Knowledge of skilled birth attendants		
Appropriate knowledge	121	53.8
Inappropriate knowledge	104	46.2
Weekly delivery cases per individual (mean ±SD)		4.04 ±2.70

The main concern of midwives was lack of authority to make decisions. For example, initiating necessary early referral of laboring mothers in case of complication prediction is often delayed due to systemic processes. An experienced midwife said *“we don’t have enough power to decide on timely and appropriate referral of laboring mother for higher and better management*. *Em…Yea*… *referral issues are expected to handle by health officers in the health centers*. *However*, *this leads unnecessarily delay until the responsible person called up and arrange referral slip which in turn again provoking further complication and death”* (FGD participant, degree midwife).

In addition, this study specified that fear of legal issues was an important barrier to satisfaction of SBAs. One health center director described *“Since the safety of mother and newborn currently is very momentous for all providers and policy makers*, *many decisions are made based on panic legal issues*, *mixing up of politics and health care*. *Everyone in the health system is terribly afraid of litigation”* (KII participant, Health center director).

### Facility characteristics

Eighty five percent of the participating health facilities were health centers. Eight out of ten health facilities did not have a regular staff rotation policy. 62.5% (n = 25) of the primary health care level facilities did not introduce any maternal and newborn health quality improvement initiative. With respect to facility readiness, more than half 21(52.55%) of the facilities were assessed as adequately ready [**Table 5].**

**Table 5:** Facility characteristics in Northern Ethiopia, 2019 (n = 40).

Variables	Number	Percentage
Facility Type		
Health Center	34	85.0
Primary Hospital	6	15.0
Facility has maternal, perinatal/neonatal death surveillance & responding (MPNDSR)		
Yes	29	72.5
No	11	27.5
Facility had regular staff rotation policy		
Yes	8	20.0
No	32	80.0
Maternal Newborn Health collect the data regularly		
Yes	30	75.0
No	10	25.0
Facility has mobile data internet access		
Yes	37	92.5
No	3	7.5
Maternal Newborn Health quality improvement initiative		
Yes	15	37.5
No	25	62.5
Facility Readiness		
Inadequately ready	19	47.5
Adequately ready	21	52.5

### Quality of process of routine childbirth care signal functions

**Fig 1:**
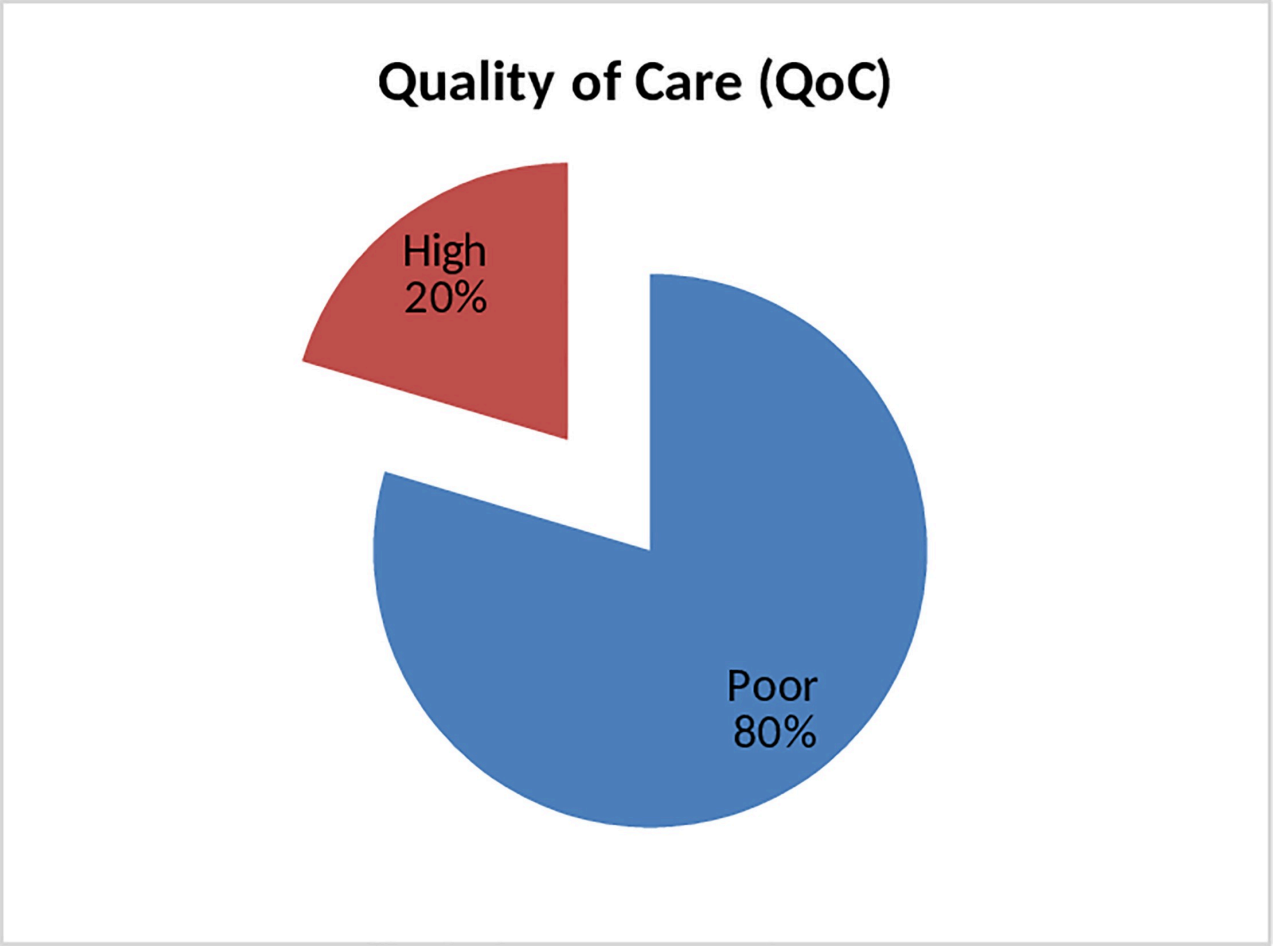
Quality of routine childbirth care signal functions.

This study showed that two out of ten (n = 181) mothers received high quality of routine child birth care signal functions in Tigray region, northern Ethiopia **(Fig 1).**

### Barriers for quality of routine childbirth care signal functions

Results of the fully adjusted regression analysis (Table 6) reveal that facility type, regular staff rotation, facility-based maternal and newborn health quality improvement initiatives, provider training on compassionate respectful maternity care, client flow for delivery, mentorship opportunities, and evidence of providers’ satisfaction significantly impacted on quality of routine childbirth care signal functions.

**Table 6:** Linear regression analysis of predictors on quality of routine childbirth care signal functions in primary health facilities of Tigray, Northern Ethiopia, 2019.

Variables	Univariate Analysis			Multivariate Analysis		
	Β	95%CI	P-value	β adj.	95%CI	P
Maternal residence						
Rural	Ref					
Urban	0.15	(-0.34,0.64)	0.552			
Maternal education						
No formal education	Ref					
Elementary school	-0.17	(-0.67,0.33)	0.501			
Secondary school and above	-0.20	(-0.71,0.31)	0.443			
**Mothers involved in decision of their care**						
No	Ref			**Ref**		
Yes	1.17	(0.64,1.71)	0.000	**0.92**	**(0.38,1.47)**	**0.001**
Estimated walking time to the nearest health facility						
30 minute and below	Ref					
Greater than 30 minutes	-0.11	(-0.59,0.38)	0.664			
ANC visit for current pregnancy						
No	Ref			Ref		
Yes	0.88	(0.07,1.68)	0.034	0.62	(-0.19,1.42)	0.134
Birth preparedness and complication readiness (BPCR)						
No	Ref			Ref		
Yes	0.29	(-0.19,0.76)	0.233	-0.05	(-0.53,0.43)	0.839
Length of labor in hours	-0.13	(-0.20,0.15)	0.601			
Mode of Delivery						
Spontaneous (SVD)	Ref			Ref		
Instrument Delivery	-0.72	(-1.41,-0.04)	0.038	-0.64	(-1.31,0.04)	0.064
Time women stay at the facility following a normal delivery						
< 6hours	0.06	(-0.41,0.53)	0.800			
6–24 hours	-0.21	(-0.70,0.29)	0.414			
>24 hours	Ref					
Parity/deliveries	-0.08	(-0.17,0.01)	0.075	-0.02	(-0.14,0.09)	0.694
Number of pregnancies/Gravidity	-0.16	(-0.28,-0.04)	0.007	-0.06	(-0.23,0.11)	0.460
History of abortion						
No	Ref			Ref		
Yes	-0.63	(-1.11,-0.16)	0.009	-0.46	(-0.96,0.05)	0.075
History of stillbirth						
No	Ref			Ref		
Yes	-0.65	(-1.38,0.08)	0.081	-0.44	(-1.21,0.33)	0.261
Allow your partner to enter to the delivery room						
No	Ref					
Yes	0.02	(-0.47,0.52)	0.926			
**Facility Type**						
Health Center	Ref			Ref		
Primary Hospital	**-1.63**	**(-4.11,0.85)**	**0.192**	**1.27**	**(0.80,1.84)**	**0.001**
Facility has MPNDSR						
No	Ref					
Yes	1.07	(-0.92,3.07)	0.284			
**Facility had regular staff rotation policy**						
No	Ref			Ref		
Yes	**1.66**	**(0.54,3.85)**	**0.135**	**2.19**	**(0.01,4.31)**	**0.019**
Facility has mobile data internet access						
No	Ref					
Yes	-1.75	(-0.14,1.94)	0.303			
**Maternal and newborn health quality improvement initiative**						
No	Ref			Ref		
Yes	**0.72**	**(-2.58,1.14)**	**0.437**	**1.58**	**(0.26,3.43)**	**0.001**
Facility readiness	-0.24	(-0.55,0.11)	0.178	1.23	(0.81,2.04)	0.247
Age of the provider	-0.02	(-0.08,0.03)	0.407			
Provider work experience	0.02	(-0.08,0.04)	0.500			
Sex of provider						
Male	Ref					
Female	0.09	(-0.72,0.91)	0.833			
SBAs’ highest level of education						
Diploma	Ref			Ref		
Degree and above	0.66	(-1.42,0.90)	0.088	-0.69	(-1.56,0.17)	0.115
Educational program attended						
Generic	Ref			Ref		
Upgrade	-0.71	(-1.33,-0.09)	0.024	0.003	(-0.82,0.81)	0.995
Professional cadre						
Midwife	0.54	(-0.14,1.21)	0.122	0.03	(-1.18,1.23)	0.963
Nurse	0.49	(-0.37,1.35)	0.263	-0.49	(-1.79,0.82)	0.463
Health officer and MD	Ref			Ref		
In the last 2 years received basic emergency obstetrics training						
No	Ref					
Yes	0.40	(-0.38,1.18)	0.315			
**Compassionate respectful maternity care training**						
No	Ref			Ref		
Yes	**-0.06**	**(-0.85,0.74)**	**0.893**	**0.08**	**(0.07,0.88)**	**0.021**
Quality improvement training						
No	Ref			Ref		
Yes	-0.39	(-1.37,0.59)	0.434			
**Client flow for delivery**	**-0.21**	**(-0.35,-0.07)**	**0.003**	**-0.19**	**(-0.34,-0.04)**	**0.012**
Legal issues fear to make decision in your daily basis care						
No	Ref					
Yes	-0.24	(-1.22,0.74)	0.625			
**Receive a clinical mentorship**						
No	Ref			Ref		
Yes	**-0.08**	**(-0.84,0.69)**	**0.842**	**0.02**	**(0.01,0.78)**	**0.049**
Receive support supervision						
No	Ref			Ref		
Yes	0.86	(0.04,1.67)	0.040	-1.23	(-2.43,1.98)	0.768
Had regular case presentation (morning session)						
No	Ref					
Yes	0.37	(-0.40,1.14)	0.347			
Having challenge in providing intra-partum, and immediate postpartum care						
No	Ref					
Yes	0.37	(-0.43,1.17)	0.365			
Motivation of HCPs	0.16	(-0.30,0.62)	0.493			
**Satisfaction of HCPs**	**0.18**	**(0.05,0.31)**	**0.005**	**0.16**	**(0.03,0.29)**	**0.013**
Knowledge of HCPs	-0.02	(-0.09,0.05)	0.630			

NB: β adj is adjusted β; P is P-value, CI confidence interval

Having one unit increase in maternal and newborn health care quality improvement initiatives in the facility, the quality of routine childbirth care signal functions provision increased significantly (β = 1.58, 95% CI: 0.26, 3.43; p = 0.001).

Every one-unit change in level of facility type (i.e., from health center to primary hospital) resulted in 1.27 increase in provision of QoC (β = 1.27, 95% CI: 0.80, 1.84; p = 0.001).

Due to change in receiving compassionate respectful maternity care training of providers in the last two years, the provision of high quality of child birth care signal functions increased significantly (β = 0.08, 95% CI: 0.07,0.88; p = 0.021). Facilities that had a staff rotation policy in place for maternal and newborn health units (more than one rotation a year) were 2.19 times more likely to provide QoC compared to facilities without staff rotation policies (β = 2.19, 95% CI: 0.01, 4.31; p = 0.019).

Every unit increase in receiving clinical mentorship lead to in 0.02 increases in the provision of QoC. For every one unit increase in involving of mothers to their care decisions, resulted in 0.92 increases in received high QoC during their routine childbirth care functions. Similarly, one unit increase in client flow for delivery of the provider resulted in 0.19 decrease in the provision of QoC (β = 0.19, 95% CI: -0.34, -0.04; p = 0.012). For every one unit increase in provider job satisfaction, the provision of QoC increased significantly (β = 0.16, 95% CI: 0.03, 0.29; p = 0.013).

According to the qualitative research findings, work related burnout, gap between providers’ skills and knowledge, being fear of litigation, poor motivation schemes and issues related to their retention, shortage of SBAs mainly midwives, lack of authority to make decisions, unable to translate training into practice and unavailability of adequate medications and necessary equipment were important reasons for poor quality of care during childbirth and immediate postpartum care. These issues are reflected in a series of quotations highlighted herein:

While poor motivation and satisfaction pressures due to low salaries

and allowance were found to greatly affect the care SBAs delivered,

they were not the only principal barriers. Fear of law suit, shortage

of human resource, unhygienic infrastructure and inadequate

availability of medicine and supplies impact up on quality practice

(KII participant, unit head of maternity ward).

… heavy work load leads providers’ burn out resulting from insufficient

and can lead to poor performance, inappropriate behavior and attitude.

In addition, limited proper capacity building devices and incompetent

SBAs remain serious barriers to provide quality of maternity care

*services in Tigray* (KII participant, Woreda MCH expert).

Despite much training conducted so far, whatever their role is

significant in improving knowledge and skill of providers, but should

have to be supplemented through coaching and mentoring which in

turn increased their level of confidence in delivering services and

enabled them to increase adherence to good practice and

*standards*.” (FGD participant, diploma midwife)

Almost all SBAs have a fear of litigation in provision of maternal and

newborn care services. Thus, have a legal protection of SBAs in terms

of medical indemnity insurance is important to apply their highest

level potential in reducing unnecessary referral and averting maternal and

*newborn death*.(KII participant, hospital medical director)

Another identified reason was poor communication between provider and parturient women and capacity of providers to adhere to standards (example: Partograph and active management of third stage of labor). A senior midwife said *“adherence to standard guidelines and an institutionalization of World Health organization safe childbirth checklist is very poor” (*FGD participant, degree midwife).

## Discussion

This study examined the quality of and barriers to routine intra-partum and immediate postpartum care functions among primary level health care facilities in Tigray regional state in northern Ethiopia using a mixed method approach. We found low QoC (only one out of five mothers received high QoC) overall. Primary hospitals, facilities which promote staff rotation, facilities having maternal and newborn health quality improvement initiatives, involvement of mothers in care decisions, training on compassionate respectful maternity care, client flow for delivery service, mentorship and providers’ satisfaction were identified as significant predictors of QoC. This finding is complemented through the qualitative results that emphasizes work related burnout, gap between providers’ skills and knowledge, lack of enabling working environment (fear of litigation and lack of authority to make decisions), poor motivation scheme and issues related to retention, poor provider caring behavior, and unable translate training into practice were important reasons for poor QoC during the delivery and immediate postpartum care period. This is lower compared to study reports done in some Sub-Saharan Africa countries [31–33]. However, this finding is slightly higher compared to a study conducted in Tanzania (14%) [34]. These variations may be due to differences in measuring standards between studies, timing of data collection, study participants included, type of health facilities, or a combination of these factors. This implies that SBAs are not adhering to standard guidelines, neglecting services to mothers and newborns, missing the most essential basic interventions, or have poor caring behaviors and skills to provide the routine childbirth care functions.

We observed a positive significant association between QoC of childbirth and staff rotation policy within maternal and newborn continuity of care units. The providers who had staff rotation policy appear best suited for provision of QoC without feeling of professional fatigue. This result is supported by study done in china [35]. This suggests that staff rotation may also reduce staff burnout and allow providers to improve their provision of quality of maternity care services. In the current study, mothers who involved to their care decisions during childbirth were more likely to receive high quality of care; this study result is consistent with studies done in Eretria [36] and in accordance with the Lancet’s global quality agenda [37]. Clearly the provision of adequate information and time for women to make informed decisions about their care and treatment in partnership with their healthcare professionals is a pivotal component of standards of maternity care services, which, in turn, increased trust and confidence in receiving continuous support, and ease of communication. One possible reason could be the majority of health providers were not attending to what women want or expect and giving priority to conducting the procedures before asking permission in advance, which is an emerging concern in Ethiopia. This align with a previous study which emphasized, with increasing service utilization, the importance of optimal interpersonal communication and involvement of mothers in decision making is likely to be a crucial dimension to maintain or increase the quality of health services [38].

Type of facility was significantly affected the quality of childbirth care provision. Those providers who are in primary hospitals were more likely to provide QoC compared to those at health centers. This result is consistent with study done Swedish [39] and Nigeria [40]. The explanation for this might lie in hospitals hosting senior staff, with many of them potentially affiliated with teaching institutions. The healthcare workers in such institutions will continually get a chance of updating their knowledge during ward round, bedsides with students, and via a series of seminars that are usually organized as a protocol of the institution. This implies that experience sharing of health centers from their catchment hospitals through regular mentoring program could enable the providers to provide high QoC services. Furthermore, our findings revealed that clinical mentorship leads to increase in the provision of high QoC of routine childbirth care signal functions.

We also found that factors at the provider level, rather than the facility level, seem to influence quality of routine childbirth care. This finding is comparable with evidence from a systematic review that outlined how several individual providers’ factors (incompetency and negative behaviors and inadequate number of staffs) affect QoC [41]. To provide QoC for laboring women and newborns in health-care facilities primarily requires appropriate staffing with high competency and motivation and with the minimum availability of essential physical resources [28].

This result further showed that delivery caseload is negatively associated with quality of care. Similarly, providers who had high numbers of deliveries were found to be less likely to provide QoC [42], which was further corroborated in studies done in Malawi [43] and other sub-Saharan African countries [21, 23] This can account for significant inequity in workloads for staff in different facilities and indeed in different units within the same facility. Participants in this study reported significant increases in the number of deliveries, mirroring the sharp increase in preference for facility-based delivery; however, there has not been a parallel increase in the number of staff to attend these women.

Other findings showed that health care providers trained on compassionate respectful maternity care were significantly associated with provision of high QoC. This result was in line with studies done in Malawi [44] and Ghana [45]. This might be due to lack of exposure to caring behaviors and poor communication between providers and clients, lack of regular updates in training, and minimal certification processes before graduation contributing to poor competencies in maternity and newborn care practice. Thus, simulation based routine and continual compassionate respectful maternity care training need to be organized for improvement of providers’ caring behaviors to minimize negative behaviors and increase their competency to adhere to standards.

Similarly, in this study, the provision of QoC has a direct relationship with providers’ job satisfaction. This is congruent with the studies done in countries of Afghanistan [46] and Pakistan [47]. This indicates that, although there has been increased interest among researchers and policymakers in identifying and implementing effective solutions to address SBAs directed motivation strategies in remote and rural areas in recent years, the current evidence available to guide policymakers on adoption and adaptation of specific retention strategies remains quite limited [48]. Thus, more attention needs to be given to develop interventions and strategies that directly enhance provider satisfaction and retention mechanisms in various contexts to improve QoC. Moreover, almost all SBAs have a fear of litigation in provision of maternal and newborn care services which is strongly suggestive of the urgent need to have a legal protection of SBAs through medical indemnity insurance in Tigray region.

Although inputs or facility readiness should serve as a foundation for high-quality care, our study did not suggest the existence of inputs necessary for providing better care within the existing infrastructure. This finding is similar to prior studies [5, 9, 49] which found that increased availability of inputs for delivery care are poorly correlated with provision of evidence-based care or explained an insignificant fraction of increased QoC delivered to women and newborns in need. This finding implies that, unless providers translate knowledge/evidence into practice, having well-equipped facilities might not often guarantee to provide high quality care and vice versa.

As a limitation of this study, we could not relate the outcome variable which is QoC during the routine childbirth and immediate postpartum period with those near miss mothers and deaths since many mothers in primary level facilities were immediately referred to higher facilities for better management. In case where such complications arise, the provider might use different standards in managing the complications other than for the normal birth. Therefore, to minimize this variability, we excluded the mothers with complications and only mothers with normal birth were interviewed. Hence, it is necessary to consider those limitations while interpreting the findings and conducting further research in general and in tertiary hospitals among those outlier mothers would be more plausible. However, this study had much strength. Quality of care was assessed using context based validated indicators which provide a more detailed picture of the state of QoC in the process of childbirth. In addition, women’s exit interviews, shortly following delivery, but prior to facility discharge might have shortened recall time and yielded more clarity in the results.

## Conclusions

There is poor QoC during the intra-partum and immediate postpartum period in primary level facilities of Tigray, Northern Ethiopia. Primary hospitals, facilities which promote staff rotation, facilities having maternal and newborn health quality improvement initiatives, maternal inclusion in decisions related to their care, training on compassionate respectful maternity care, mentorship and providers’ satisfaction were linked with significant increases in QoC. However, client flow for delivery is negatively associated with QoC. This finding was complemented by the second phase (i.e, the qualitative approach) that revealed work related burnout, gap between providers’ skills and knowledge, lack of enabling working environments (fear of litigation), poor motivation scheme and issues related to retention, poor provider caring behaviors, lack of translations of training into practice, mismatch between the number of provider and facility client flow for delivery and lack of essential medicines and supplies were major bottlenecks in the provision of timely and quality obstetric care, which has a significant impact on maternal and neonatal outcomes.

Therefore, efforts must be made to improve the QoC through experience sharing of health facilities within their respective catchments, and have a legal protection of SBAs in terms of medical indemnity insurance. More attention and thoughtful strategies that match providers to workload, coupled with targeted efforts to support providers’ satisfaction and health-care worker performance and retention, are necessary to mitigate the effects of working in this context and to improve the quality of obstetric care.

Initiating or providing regular catchment-based mentoring and adopting quality improvement initiatives for skilled providers are essential in order to increase adherence to good practices and standards. Furthermore, having staff rotation within maternal newborn units (more than one rotation a year) may minimize work related burnout which, in turn, leads to improved QoC in our context.

## Supporting information

S1 Appendix The qualitative research guide both FGD and key informant interview to Regional head/Woreda/Director of hospital, health center and senior SBAs mainly Midwives. (DOCX)

S2 Appendix Detail measurement tool of potential QoC indicators during child birth and immediate postpartum period through the Principal Component Analysis (PCA). (DOCX)

S3 Appendix Detail measurement tool of providers satisfaction through the Principal Component Analysis (PCA). (DOCX)

S4 Appendix Basic emergency obstetrics related standard knowledge questionnaire assessment tool for SBAs working on intra-partum & immediate postpartum care. (DOCX)

S1 File (SAV)

## List of abbreviations

KII: key informant interviews
PCA: Principal Component Analysis
QoC: Quality of Care
SARA: Service Availability and Readiness Assessment survey
SBA: Skilled Birth Attendant
WHO: World Health Organization
