# Supplementary material for: Quality of and barriers to routine childbirth care signal functions in primary level facilities of Tigray, Northern Ethiopia: Mixed method study
Source: PLoS One. 2020 Jun 12;15(6):e0234318. doi: 10.1371/journal.pone.0234318 (PMC7292403; doi:10.1371/journal.pone.0234318)
Supplement: S1 Appendix — (DOCX) [file pone.0234318.s001.docx]

**MEKELLE UNIVERSITY, COLLEGE OF HEALTH SCIENCES, SCHOOL OF PUBLIC HEALTH,**

**QUALITATIVE RESEARCH GUIDE**

**1. Key informant interview to Regional Head/Woreda/Director of Health Center**

Dear Participant,

My name is ____________ and I am a member of research team from Mekelle University, College of Health sciences. We are conducting a research study on improving quality of intrapartum and immediate postpartum care Tigray Region. You are identified as someone who are coordinating and managing this Region/Woreda or Health facility and would be willing to briefly talk with me about your perceptions on Quality of Care. I would like to talk with you for 30-45 minutes.

This conversation will be recorded for research accuracy and transcription. Your participation in this research is voluntary. You may withdraw your consent and stop participation at any time without penalty. Your comments during this interview will be kept confidential in final reports. Only the researchers (PI) will have access to data tagged with organizational sources, which will be stored on a secured server. While there are no direct benefits to you, we hope to gain more knowledge on how to improve quality of health care services.There are no risks associated with this research. Are there any questions about what I have just explained?

Are you willing to participate in this interview? Yes No

Interviewee sig ________________________Date of interview _________________

Interviewer Name__________________________signature________________________

Supervisors name __________________________signature_____________________

I thank you in advance for your willingness to help with this research!!!

| **Identification and Socio demographic factors** | |
| --- | --- |
| Woreda |  |
| Facility name |  |
| Religion |  |
| Marital status |  |
| Educational level |  |
| Profession |  |
| Position |  |

1. How do you understand Quality of maternal and newborn health care? Specifically the care provided during intrapartum and immediate postpartum period?
2. What is/are the major challenge/s that makes health care providers to provide a poor quality of care in health facility especially during the intrapartum and postpartum?
3. What initiatives are there in your organization to improve quality of care?
4. In your opinion, what could be done to improve quality of intrapartum and immediate post-partum care?

**2. Focus group discussion to Skilled Birth Attendants**

Dear Participants,

My name is ________________. I am a member of research team from Mekelle University, College of Health science and I would like to thank you for finding time to come and attend this discussion. It is going to be a discussion on to assess challenges and opportunities of quality of care in relation to evidence based obstetric care practices at intrapartum and immediate postpartum period in primary health care facilities and information from our discussion will be used in making future improvements of the Quality of care. The information from the discussion will be treated with confidentiality.The tape recorded information will not be accessed by anyone except the researchers and it will only be used for this research purpose.

| S. No | Code | Signature |
| --- | --- | --- |
| 1 |  |  |
| 2 |  |  |
| 3 |  |  |
| 4 |  |  |
| 5 |  |  |
| 6 |  |  |
| 7 |  |  |
| 8 |  |  |
|  |  |  |

**Theme 1: Factors related to person providing care: (ኣርእስቲ 1፡ምስ ግልጋሎት ዋሃቢ ዝተትሓሓዙ ሕቶታት)**

1. How do you manage women in labour who came to this labor ward?(**Probe:** availability of guidelines and their use, monitoring of maternal and fetal condition, Parthograph, episiotomy, AMTSL…)

(ከመይ ገይረኩም ኢኩም ናብዚ መዋለዲ ክፍሊ ዝመፃ ወላዳት ትሕክምወን?(መብራህርሂ፡ህላወ ሓፈሻዊ መምርሒን ኣጠቓቅምኡን፣ክትትል ኩነታት ኣዶን ህፃን፣ፓርቶግራፍ፣ምቕዳድ ፍንዶት፣ሳልሳይ ደረጃ ክንክን ሕርሲ…)

1. When a woman comes with normal labour, what are some of the things that you will encourage her to do?**(Probe:** empting of the bladder, movement during labour, labour position, oral and Iv fluids)

(ኣዶናብወሊድኣብእትመፀሉእዋን፤እንታይዓይነትነገራትክተከናውንተበራታትዕዋ?(መብራህርሂ፡ሽንቲምሻን፣ኣብእዋንሕርሲምንቕስቃስ፣ኣቀማምጣኣዶ፣ብኣፍንብሰራውርደምንዝውሰድፈሳሲ)

1. Why intrapartum care providers provide poor QoC or use ineffective practices during child birth? (**Probe:** Fundal pressure, enema, routine aminotomy, restriction of oral fluids and food and liberal episiotomy).(ንምንታይእዮምባዓልሞታትኣብእዋንወሊድዘይምከርተግባርዘካይዱ? (መብራህርሂ፡ከብዲጥንስቲኣዶምፅቃጥ፣ምሕፃብማዓንጣ፣ምፍሳስፈሳሲ፣ማይንምግብንምክልካል፣ልቂምቕዳድፍንዶት)

**Theme 2: Quality Improvement initiatives and lessons (ኣርእስቲ 2፡ ፅሬት ግልጋሎት ንምምሕያሽ ተበግሶታትን ትምህርትን)**

1. How do you understand Quality of Care, what are the components/ indicators?

ኣወሃህባፅሬትግልጋሎትከመይትርደኦ/ትገልፆ፤እንታይዓይነትክፋላት /ሓበርቲኣለውዎ?

1. Do you have any quality MNH improvement initiatives in your organization? **(Probe:** catchment based mentorship, Support supervision and others)ኣብ ትካልኩም ዝኮነ ዓይነት ኣወሃህባ ፅሬት ግልጋሎት ኣዴታትን ህፃናትን ከመሓይሹ ዝክእሉ ተበግሶታት ኣለዉ ዶ? (መብራህርሂ፡ኣብ ከባቢኻ መሰረት ዝገበረ ድጋፋዊ ክትትል፣ድጋፋዊ ዑደትን ካልኦትን)
2. Which type of the quality improvement initiative do you think suites in bring quality to your service specifically to childbirth?

ን ትካልኩም ኣንታይ ዓይነት ፅሬት ምምሕያሽ ተበግሶ እዩ ምስ ምምሕያሽ ፅሬት ኣወሃህባ ግልጋሎት ዝስማማዕ?

1. What are the challenges in providing quality of intrapartum and immediate postpartum care?(Probe: workload, Training and mentoring)ኣብ ኣወሃህባ ፅሬት ግልጋሎት ኣብ እዋን ወሊድን ወድውኑ ድሕሪ ወሊድ ዝዋሃብ ክንክን እንታይ ዓይነት ፀገማት ኣለዉ?(መብራህርሂ፡ስራሕ ፃዕቂ፣ስልጠናን ድጋፋዊ ክትትልን)
2. What will you recommend to improve quality of intrapartum and immediate postpartum care **(Probe:** pre service/ in-service trainings, catchment based mentorship, support supervision etc…)

እንታይ እንተዝግበር ትብል ኣብ ምምሕያሽ ፅሬት ኣወሃህባ ግልግሎት ኣብ እዋን ወሊድን ቁልጡፍ ድሕሪ ወሊድ ዝዋሃብ ክንክን?(ቅድሚ/ኣብ ስራሕ ዝዋሃብ ስልጠና፣ኣብ ከባቢኻ መሰረት ዝገበረ ድጋፋዊ ክትትል፣ድጋፋዊ ዑደት)

**Theme 3: Factors related to laboring women: (ኣርእስቲ 3፡ምስ ተገልጋሊት ኣደ ዝተትሓሓዙ ሕቶታት)**

1. What are the client side challenges in adopting evidence based obstetric care practice?ብወገን ተገልጋላይ ኣብ ምትግባር ብመረዳእታ ዝተደገፉ ስራሕቲ ብዛዕባ ጥንስን ሕርሲን ከምኡ እውን ቅልጡፍ ድሕረ ወሊድ ክንክን ዘለዉ ፀገማት እንታይ እዮም?
2. Why don’t mothers allow their companion/partner to enter to delivery room?

ኣዴታት ኣብ እዋን ወሊድ ንምንታይ እየን ሓጋዚ/ባዓል ገዝአን ናብ ዝወልዳሉ ቦታ ክኣቱ ዘይደልያ?

1. Did mothers be involved in decision making for the type of care they received? If yes, what are the facilitators for decision making? If not, what are the reasons for not deciding?

ኣዴታት ኣብ እዋን ወሊድ ዝዋሃበን ግልጋሎት ባዕለን ይውስና ዶ? እወ እንተኾይኑ፤ንኽውስና ዝግብርወን ምክንያታት እንታይ እዮም?ኣይፋሉን እንተኾይኑስ፤ምኽንያቱ እንታይ እዮም?

1. What do you suggest us to adopt evidence based obstetrics care towards mothers side?ብመረዳእታ ዝተደገፉ ስራሕቲ ብዛዕባ ጥንስን ሕርሲን ከምኡ እውን ቅልጡፍ ድሕረ ወሊድ ክንክን ኣብ ምትግባር ብ ወገን ተገልጋሊት ኣዶ እንታይ ተማኹርና?

**Theme 4: Factors related to health system (ኣርእስቲ 4፡ ምስ ስርዓተ ጥዕና ዝተሓሓዙ ሕቶታት)**

1. Do you have to date standards or guideline on quality of obstetrics care including evidence based obstetric care practices, if yes have you used it in your daily practice? If not used it why? And if there is not available why?

ዝተማሓየሹ/እዋናዊ መመርሕታት ኣብ ኣዋሃህባ ፅሬት ግልጋሎት ጥንስን ሕርስን ብተወሳኺ ብመረዳእታ ዝተደገፉ ስራሕቲ ብዛዕባ ጥንስን ሕርሲን ከምኡ እውን ቅልጡፍ ድሕረ ወሊድ ክንክን ኣለውኩም ዶ?መልስኹም እወ እንተኾይኑ ኣብ ማዓልታዊ ስራሕኩም ትጥቀምሎም ዶ?እንድሕር መልስኹም ኣይፋሉን ኮይኑ ንምንታይ ኢኩም ዘይትጥቀምሎም?እንድሕር ዝተማሓየሹ መምርሕታት ዘይሃልዮም ንምንታይ?

1. Do you think that you have adequate capacity building mechanisms? **(Probe:** any quality MNH care training, catchment based mentorship, support supervision and others )

እኩል ዝኮነ ናይ ሓይሊ ሰብ መዕበይ ዓቅምታት ኣለዉ ዶ ኢልካ ትሓስብ?(መብራህርሂ፡ዝኾነ ይኹን ፅሬት ኣዋሃህባ ግልጋሎት ኣብ ጥንስን ሕርስን፣ከባብያዊ መሰረት ዝገበረ ድጋፋዊ ክትትል፣ድጋፋዊ ዑደትን ካኦትን)

1. What type of challenges you have faced during these capacity building activities?

ኣብ እዋን ዓቅሚ ምዕባይ ተግባራት እንታይ ዓይነት ፀገማት የጋጥምኹም?

1. What do you suggest us to adopt evidence based obstetrics care/quality of care in relation to health system?

ብመረዳእታ ዝተደገፉ ስራሕቲ ብዛዕባ ጥንስን ሕርሲን ከምኡ እውን ቅልጡፍ ድሕረ ወሊድ ክንክን ኣብ ምትግባር ምስ ስርዓተ ጥዕና ተተሓሒዙ እንታይ ተማኹርና?
