# Supplementary material for: Quality of and barriers to routine childbirth care signal functions in primary level facilities of Tigray, Northern Ethiopia: Mixed method study
Source: PLoS One. 2020 Jun 12;15(6):e0234318. doi: 10.1371/journal.pone.0234318 (PMC7292403; doi:10.1371/journal.pone.0234318)
Supplement: S2 Appendix — (DOCX) [file pone.0234318.s002.docx]

**Checklist on Potential Quality of intrapartum and immediate post-partum care indicators for women experiencing normal deliveries**

(ክፍሊ ኣርባዕተ፡ ኣብ እዋን ምስንባት ዝምላእ ናይ ፅሬት ኣገልግሎት መቆፃፀሪ ዝርዝር)

| **S/No**  **(ተ.ቑ)** | **Task or Activity** (ስራሕቲ / ንጥፈታት) | **Correctly Performed** ብትክክል ተሰሪሑ | | | | | | | | **Skip /** ናብ ቀፃሊ ሕቶ ይዘለል | |
| --- | --- | --- | --- | --- | --- | --- | --- | --- | --- | --- | --- |
|  |  | Yes/እወ | | No/ኣይሉን | | | DK/ኣይፈልጦን | | |  |  |
| 401 | Did the provider greet you and her companion (if present) respectfully (እቲ በዓል ሙያ ንዓኪ ወይ ምሳኪ ንዝመፀ ቤተሰብ ብዝግባእ ክብሪ ዝተማልአ ሰላምታ ሂቡኪ ዶ? | | 1 | | 0 | | | 8 | | |  |
| 402 | Did the provider actively listen you (able to respond for your concern, allocate adequate time to talk) (እቲ በዓል ሞያ ንሓሳብኪ ብፅሞና ኣዳሚፅዎዶ? | | 1 | | 0 | | | 8 | | |  |
| 403 | Were you allowed to have a support person (companion) with you during your labor (ሓጋዚኺ /ባዓል ገዛኺ ኣብ መፅንሒ ክፍሊ ምሳኺ ንክህልይ ፈቒዶምልኪ ዶ? | | 1 | | 0 | | | 8 | | |  |
| 404 | If not checked before, did someone offer youan HIV test (ንእንድሕር ዘይተመርሚርኪ ክትምርመሪ ተሓቲትኪ ዶ?) | | 1 | | 0 | | | 8 | | |  |
| 405 | While you were at this facility fo birth of your baby,did any one test you for HIV? (ተመርሚራ ዶ?) | | 1 | | 0 | | | 8 | | |  |
| 406 | Did someone take your temperature (ሙቐት ተለኪዕኪ ነይርኪ ዶ?) | | 1 | | 0 | | | 8 | | |  |
| 407 | Did someone take blood pressure (ፀቕቲ ደም ተለኪዕኪ ነይርኪ ዶ?) | | 1 | | 0 | | | 8 | | |  |
| 408 | Did the provider washes his/her hands with soap and water or uses antiseptic before examining you / በዓል ሞያ ቅድመን ድሕሪን ዝኾነ ዓይነት ምርመራ ሕክምና ምግባሩ ኢዱ ተሓፂቡ ዶ? | | 1 | | 0 | | | 8 | | |  |
| 409 | Did the provider perform vaginal examination (ብልዕታዊ ምርመራ ገይሩልኪ ዶ?) | | 1 | | 0 | | | 8 | | |  |
| 410 | Were you allowed to get up and walk or ambulate around while you were in labor? (እቶም ሰብ ሙያ ጥዕና ኣብ ቀዳማይ ደረጃ ሕርሲ እናሃለኪ ንክትንቀሳቐሲ ይፈቅዱልኪ ዶ ነይሮም? | | 1 | | 0 | | | 8 | | |  |
| 411 | Were you allowed to drink liquids or eat any food while you were in labor (እቶም ሰብ ሙያ ጥዕና ኣብ ቀዳማይ ደረጃ ሕርሲ እናሃለኪ ፈሳሲ ንክትሰትይ ወይ ድማ ምግቢ ንክትበልዒ የበራታትዑኪን ይፈቅዱልክን ዶ ነይሮም? | | 1 | | 0 | | | 8 | | |  |
| 412 | Just after the delivery of your baby in the first few minutes after the delivery of your baby did any one give you a correct Active Management of Third Stage of Labor (AMTSL)/ ህፃንኪ ድሕሪ ምውላድ ርእሱ ቅልጠፍን ፅፉፍን ኣታኣላልያ ሳልሳይ ደረጃ ሕርሲ ብዝምልከት፡   1. Correctly administer uterotonic (timing, doseand route)/ማህፀን መኮምተሪ ዝበሃልመድሓኒት ብትኽክል (ኣብ ሰዓቱን፣ ቦትኡን መጠኑ ብዝሓለወን መንገዲ) ተዋሂቡኪ ዶ? | | 1 | | 0 | | | 8 | | |  |
| 413 | Did the provider make a follow-up, monitoring of your vital signs and amount of external blood loss immediately after delivery/ ድሕሪ ምውላድኪ መድመይቲ ከየጋጥሙክን፣ መሰረታዊ ኩነታት ጥዕናኪ ጥቡቕ ክትትል/ ፀቕጢ ደም፣ ዉቅዒት ልቢ/ ገይሩልኪ ዶ?   1. Check for bleeding (ምርግጋፅ ድሕሪ ወሊድ መድመይቲ) | | 1 | | 0 | | | 8 | | |  |
|  | 1. Was the woman given any pain relief medication during labor, delivery or immediate postpartum? (ኣብ እዋን ወሊድ ቃንዛ መዐገሲ መድሓኒት ተዋሂቡኪ ዶ?) | | 1 | | 0 | | | 8 | | |  |
| 414 | Components of essential new-born care, Was your provider፡   1. Properly dried off and wrapped of your new-born /ነቲ ናፅላ ህፃንኪ ምሰተወለደ ብግቡእ ፀሪጉ ራሕሲ ንክደርቕ ተሸፊኑ ተቀሚጡ ዶ? | |  | | 0 | | | 8 | | |  |
|  | 1. Ties or clamps and cut cord after birth / ዕትብቲናፅላ ህፃንኪብግቡእ ብምእሳር ፅሬቱ ብዝሓለወ መሳርሒ ዶ ቆሪፀምሉ? | | 1 | | 0 | | | 8 | | |  |
|  | 1. Place the baby on your chest(skin-to-skin) immediately after delivery   እቲ ናፅላ ምስተወለደ ሽዑ ንሽዑ ናብ ሑቕፊ ኣዲኡ ዶ ገይርዎ፣ ማለት ሙቐት ንኽረክብ ሰዉነት ንሰዉነት ዶ ክሑቖፍ ተገይሩ? | | 1 | | 0 | | | 8 | | |  |
|  | 1. Did you breast feed your baby with in the first hour after delivery? ህፃንኪ ኣብ ዉሽጢ ሓደ ሰዓት ጡብ ምጥባዉ ጀሚርኪሉ ዶ? | | 1 | | 0 | | | 8 | | |  |
|  | E) Apply eye care using antimicrobial drop or TTC ointment? ነቲ ዝተወልደ ናፅላኺ ናብ ዓይኑ ፀረ ባክተርያ(ቴትራሳይክልን) ሂቦምዎ ዶ? | | 1 | | 0 | | | 8 | | |  |
| 415 | Did the provider maintain privacy in providing clinical care (keeps your audio privacy, visual privacy; use of partitions, cover etc…)/ እቲ በዓል ሞያ ሕክምናዊ ክንክን ኣብ ዝህበሉ ግዘ ዉልቃዊ ክብርኪ (Privacy) ሓልይልኪ ዶ? ንኣብነት: ሰዉነትኪ ብምሽፋን፣ ናይ ምክክር ድምፆም ከይስማዕ፡ ፎቶን ቪድዮን ካብምልዓን ምቕራፅን ወዘተ ሓሊዩ ዶ? | | 1 | | 0 | | | 8 | | |  |
| 416 | Did the provider give you adequate Time, information regarding your treatment and care (about the problem, prognosis, & discharge criteria etc…)/ እቲ በዓል ሞያ ብዛዕባ እቲ ዝወሃበኪ መድሓኒትን ዝግበር ክንክን ጥዕናን እኹል ሓበሬታን ረኪብኪ ዶ? | | 1 | | 0 | | | 8 | | |  |
| 417 | Did the provider gave you appropriate counseling and Health education at time of discharge (Care provision) on the following components: እቲ ብዓል ሙያ ካብቲ ትካል ጥዕና ቅድሚ ምዉፃእኪ ኣብዞም ኣብ ታሕቲ ተዘርዚሮም ዘለዉ ነጥብታት መሰረት ብምግባር ግቡእ ኣስተምህሮን ምኽርን ሂቡኪ ዶ? | |  | |  | | |  | | |  |
|  | 1. Exclusive breast feeding & breast care/ ብዛዕባ ምጥባዉ ፀባ ጡብ ኣደ ጥራሕን ክንክን ጡብን | | 1 | | 0 | | | 8 | | |  |
|  | 1. Family Planning- birth spacing/ትልሚ ስድራን፡ ኣረሓሒቅካ ምዉላድ ብዝምልከት | | 1 | | 0 | | | 8 | | |  |
|  | 1. Immunization and other prophylaxis/ ክትባትን ፀረ ካልኦት ቅድመ ሕማማት ምክታብን | | 1 | | 0 | | | 8 | | |  |
|  | 1. Review possible complication and readiness plan, both Newborns and mother danger sign before discharge/ካብ ትካል ቅድሚ ምዉፃእኪ፣ከጋጥሙኪ ንዝኽእሉ ሓልክታት ጥዕናን ከመይ ክትምክትዮም ከም ዘለክን፣ሓደገኛ ምልክታት ዝበሃሉን ኣመልኪቱ ትልሚ ንኽትገብሪ ፈተሸ ተገይሩልኪ ዶ? | | 1 | | 0 | | | 8 | | |  |
| 418 | Did the provider practice any of the following activities during your childbirth and immediate postpartum care process? (CIRCLE ALL THAT APPLY)እቲ በዓል ሞያ ካብቶም ኣብ ታሕቲ ተዘርዚሮም ዘለዉ ተግባራት ተዋሂቦምኪ ዶ ነይሮም | | Yes | | No | | | D/K | | |  |
|  | Excessive stretching of the perineum during the second stage of labor   1. ኣብ ካልኣይ ደረጃ ሕርሲ ንብልዕትኪ ብክልቲኡ ኣፃብዕቱ ገይሩ ክሰፍሕ ምግባር/ ምግታር | | 1 | | 0 | | | 8 | | |  |
|  | 1. For your delivery, shortly before you delivered your baby, did any one cut the opening of your vagina (episiotomy) to make more room for the baby’s head?ናይቲ ህፃን ርእሲ ቶሎ ንምውላድ ምቅዳድ ብልዕቲ ብኣግባቡ ዶ ተሰርሒሉኪ? | | 1 | | 0 | | | 8 | | |  |
| 419 | Did you experience any of the obstetric complications during or after your delivery? ነዚ ህፃን ክትወልዲእንከለኪ ኣብዚ ትካል ዘጋጠመኪ ፀገም/ሓልኪ ጥዕና ነይሩ ድዩ? | | 1 | | 0 | | | 8 | | |  |
| 420 | If yes for **Q419,** at what stage of Labor & Delivery did the complication occur? መልሲ ንሕቶ ቁፅሪ 419 እወ እንተኾይኑ፣ ኣበየናይ ደረጃ ሕርሲ እዩ ክትወልዲ እንከለኪ አቲ ፀገም ዘጋጢሙኪ? | | 1. During labour including admission/ሕርሲ ጀሚርኒ ናብ ትካል እንትኣትው ጀሚሩ 2. During delivery/ኣብ ግዘ ወሊድ 3. Postpartum(before discharge)   ድሕረ ወሊድ/ ኣብ ትካል እንከለኩ | | | | | | | | |
| 421 | If yes for **Q419**, can you tell me the type of complication you face?  መልስኪ ንቁፅሪ **419** እወ እንተኾይኑ ፣ ኣየናይ ዓይነት ፀገም ጥዕና እዩ ኣጋጢሙኪ? | | 1. Hemorrhage/መደመይቲ 2. Preeclampsia & Eclampsia/ ምንፍርፋርን 3. Obstructed labour/ዝተዓገተ ሕርሲ 4. Sepsis/Infection/ረኽሲ 5. Tear/laceration/ምልሓፅ ብልዕቲ 6. Maternal death/ሞት ኣደ 7. Other (specify)ይፀሓፍ/___________ | | | | | | | | |
| 422 | Did you experience any of the neonatal complication within the health facility before discharge? ዝኾነ ዓይነት ሓልኪ/ ፀገም ጥዕና ናፅላ ህፃን ኣጋጢሙኪ ነይሩ ድዩ? | | 1 | | | 0 | | | 8 | | |
| 423 | If yes for **Q419,** what type of complication does newborn have? መልሰን ንቁፅሪ 419 እወ እንተኾይኑ፣ ኣየናይ ዓይነት ፀገም ጥዕና እዩ እቲ ናፅላ ህፃን ዘጋጠሞ? | | 1. Asphyxia/ምዕፋን/ ዘይምስትንፋስ 2. Still birth/ምዉት ምዉላድ 3. Infection/ረኽሲ 4. Early neonatal death within 24 hours/ኣብ ዉሽጢ 24 ሰዓት ሞት ናፅላ 5. Others (specify) ይገለፅ__________ | | | | | | | | |
| 424 | Were there any measure delays in needed treatment during your intrapartum and immediate postpartum care? ንክትወልዲ ናብ ትካል ካብ ዝኣተክሉ ሰዓት ጀሚሩ እስካብ ሐዚ ግልጋሎት ኣብ ምውሃብ ይዝንግዑኪ ነይሮም ዶ? | | 1. No (ኣይፋሉን) 2. Yes (እወ) | | | | | | | | |
