# Supplementary material for: Quality of and barriers to routine childbirth care signal functions in primary level facilities of Tigray, Northern Ethiopia: Mixed method study
Source: PLoS One. 2020 Jun 12;15(6):e0234318. doi: 10.1371/journal.pone.0234318 (PMC7292403; doi:10.1371/journal.pone.0234318)
Supplement: S3 Appendix — (DOCX) [file pone.0234318.s003.docx]

**Providers Satisfaction Assessment tool**

| 220 | Are you satisfied with your job? In terms of: ብዞም ኣብ ታሕቲ ዝተዘርዘሩ ነጥብታት ብስራሕኻ ትዓግብ ዶ? | Yes No |
| --- | --- | --- |
|  | **Safety Domain/ ናይ ድሕንነት ዓንዲ** |  |
|  | 1. Salary you received/ እትኽፈሎ ደሞዝ | 1 0 |
|  | 1. Overall benefit package (duty fee, hazard and free medical service etc…)   ብጠቕላላ እትረኽቦ ጥቕማ ጥቅሚ (ናይ ሓዳሪ፣ናይ ሙያ ተቓላዕነት፣ ነፃ ሕክምና ኣገልግሎት ወዘተ…) | 1 0 |
|  | 1. Annual/sick/maternity leave policy**/** ዓመታዊ /ሕማም/ወሊድ ፍቓድ ስርዓት ዝምልከት | 1 0 |
|  | 1. Flexibility in scheduling your working hours & weekends ናይ ስራሕ ሰዓትን ቐዳመ ሰናብትን ዝተጥዓዓመ ፕሮግራም ምህላው | 1 0 |
|  | 1. Staff rotation/ ናይ ስራሕ ድርሻ ምቕይያር | 1 0 |
|  | 1. Opportunity for part-time work/ ትርፊ ሰዓት ስራሕ ናይ ምርካብ ዕድል | 1 0 |
|  | 1. Current assigned tasks/ ናይ ሐዚ መደብ ስራሕኻ | 1 0 |
|  | **Social Domain /ማሕበራዊ ዓንዲ** |  |
|  | 1. Recognition from your head for your work/ ካብ ናይ ስራሕ ሓላፊካ ዝተረከበ ሽልማት | 1 0 |
|  | 1. Solidarity and collaboration among colleagues/ሓድነትን ትሕብብርን ኣብ ሞንጎ መሳርሕቲ | 1 0 |
|  | 1. Availability of medical equipment’s/supplies/ ናይ ሕክምና ናውቲን መሳርሕን ምህላው | 1 0 |
|  | 1. Satisfaction with the midwifery care given to clients/ ብትህቦ ኣገልግሎት እትረኽቦ ዕግበት | 1 0 |
|  | 1. Opportunities for social contact**/** ብማሕበራዊ ርክብ እትረኽቦ ዕድል | 1 0 |
|  | **Psychological Domain /አእምራዊ ዓንዲ** | 1 0 |
|  | 1. Opportunities for further education / ናይ ቐፃላይ ት/ቲ ዕድል | 1 0 |
|  | 1. Opportunities to participate in morning rounds/ በቢ ንጉሆ ዝካየድ ፈተሻ ሕሙማት ናይ ምስታፍ ዕድል | 1 0 |
|  | 1. Opportunity to make autonomous care decisions**/** ዓርስኻ ብዓርስኻ ወሲንካ ኣገልግሎት ኣብ ምሃብ ዕድል | 1 0 |
|  | 1. Opportunities for on job training/coaching/short term training etc. ዓቕሚ መዕበይ ሓፀርቲ ስልጠናታት | 1 0 |
|  | 1. Recognition for your work from peers/ partners/ ካብ መሳርሕትኻ ብዝተረከበ ሽልማት | 1 0 |
|  | 1. Encouragement and positive feedback from your head/ካብ ሓላፊ ዝወሃብ ሃናፂ ዝኮነ ሓሳብን ግብረ መልስን | 1 0 |
|  | 1. Opportunities to participate in midwife research**/** ኣብ ናይ ሚድዋይፈሪ ዝስራሕ መፅናዕትን ምርምርን ምስታፍ ዕድል | 1 0 |
|  | 1. Opportunities to write and publish different publications/ኣብ ምፅሓፍን ምሕታምን ዘለካ ዕድል | 1 0 |
|  | 1. Your control over conditions in your working unit/ward**/** ኣብ ናይ ስራሕ መደብካ ብዘሎ ቑፅፅር | 1 0 |
|  | 1. Institutional development plan/ ናይ ትካላዊ ዕብየት ትልሚ | 1 0 |
|  | 1. Your participation in organization decision making/ ኣብ ትካላዊ ውሳኔታት ዘለካ ተሳትፎ | 1 0 |
|  | 1. Consideration given to your opinion and suggestions for change in the work   ናይ ትካል ኣሰራርሓኻ ኣብ ምልዋጥ እትህቦ ሓሳብ ዘለካ ተቐባልነት | 1 0 |
