# Supplementary material for: Quality of and barriers to routine childbirth care signal functions in primary level facilities of Tigray, Northern Ethiopia: Mixed method study
Source: PLoS One. 2020 Jun 12;15(6):e0234318. doi: 10.1371/journal.pone.0234318 (PMC7292403; doi:10.1371/journal.pone.0234318)
Supplement: S4 Appendix — (DOCX) [file pone.0234318.s004.docx]

**KNOWLEDGE OF SKILLED BIRTH ATTENDANTS WORKING ON INTRAPARTUM & IMMEDIATE POSTPARTUM CARE ASSESSMENT QUESTIONNAIRE**

**Part I: Multiple choice questions (**Circle the correct answer from the choices given for each question**)**

1. Infection can be transmitted from clients to health care workers through:
2. Contaminated needles or other sharps instruments that pierce the health worker‘s skin
3. Splashes in the health care worker‘s eye of contaminated blood or body fluids
4. Broken skin that is exposed to contaminated blood and body fluids
5. All of the above
6. The single-most practical procedure for preventing the spread of infection is:
7. Wearing gloves
8. Wearing a mask
9. Hand washing
10. Cleaning skin with alcohol before injections
11. Rapid initial assessment should be carried out
12. Only on women who present with abdominal pain and vaginal bleeding
13. Only on women who present with abdominal pain
14. Only on women who present with vaginal bleeding
15. On all women of childbearing age who present with a problem
16. Woman who suffers shock as a result of an obstetric emergency may have
17. a weak, fast pulse
18. high blood pressure
19. normal breathing
20. a good urine output

5. The partograph is a record of

1. Labor for women who experience problems
2. The main observations made during labor
3. Only maternal well-being
4. Only fetal well-being
5. Plotting on the partograph should begin
6. In the active phase of the first stage of labor
7. In the latent phase
8. When the cervix reaches full dilatation
9. When the woman is admitted to the labor ward
10. Before applying controlled cord traction during active management of the third stage of labor
11. Oxytocin is administered intramuscularly and the attendant waits for the uterus to contract
12. The woman‘s bladder is catheterized
13. Pressure is applied to the fundus
14. All of the above
15. Active management of the third stage of labor is believed to
16. Reduce blood loss
17. Shorten the third stage of labor
18. Minimize the time at which the woman is at risk of hemorrhage
19. All of the above
20. Cervical dilation plotted to the right of the alert line on the Parthograph indicates

A. Satisfactory progress of labor

B. Unsatisfactory progress of labor

C. The end of the latent phase

D. The end of the active phase

10. Diastolic blood pressure of 90 mm Hg or more before 20 weeks of gestation is a sign of:

A. Mild pre-eclampsia

B. Chronic hypertension

C. Superimposed mild pre-eclampsia

D. Pregnancy-induced hypertension

11. Eclamptic fits may occur in the፡

A. Antepartum period only

B. Intrapartum period only

C. Postpartum period only

D. Antepartum, intrapartum or postpartum periods

12. The loading dose of magnesium sulfate is given via

- 1. IV over 5 minutes, followed by deep IM injection into each buttock
  2. IV over 5 minutes, followed by deep IM injection into one buttock
  3. IM injections
  4. IV bolus, followed by deep IM injection into each buttock

13. An antihypertensive drug should be given for hypertension in severe pre-eclampsia or eclampsia if diastolic blood pressure is

A. Between 100 and 110 mm Hg

B. 110 mm hg or more

C. 115 mm Hg or more

D. 120 mm Hg or more

14. Immediate postpartum hemorrhage can be due to

A. Atonic uterus

B. Trauma to the genital tract

C. Retained placenta

D. All of the above

15. Tears of the cervix, vagina or perineum should be suspected when there is immediate postpartum hemorrhage and

A. A complete placenta and a contracted uterus

B. An incomplete placenta and a contracted uterus

C. A complete placenta and an atonic uterus

D. An incomplete placenta and an atonic uterus

16. Postpartum hemorrhage is defined as

A. Vaginal bleeding of any amount after childbirth

B. Sudden bleeding after childbirth

C. Vaginal bleeding in excess of 300 mL after childbirth

D. Vaginal bleeding in excess of 500 mL after childbirth

17. If manual removal of the placenta is performed

A. Give ergometrine prior to the procedure

B. Give antibiotics 24 hours after the procedure

C. Place one hand in the uterus and use the other hand to apply traction on the cord

D. Place one hand in the uterus and one hand on the abdomen to provide counter

18. For repair of vaginal and perineal tears, local anesthetic should be infiltrated

A. Beneath the vaginal mucosa

B. Beneath the skin of the perineum

C. Deeply into the perineal muscle

D. All of the above

19. Factors that may predispose to postpartum infection

A. Prolonged labor and prolonged rupture of membranes

B. Frequent passing urine

C. Not wearing gloves before palpating the abdomen

D. Giving plenty of drinks during labor

20. The treatment of metritis should include

A. IV ampicillin or iv gentamicin or iv metronidazole

B. IV ampicillin, plus iv gentamicin and iv metronidazole

C. A combination of oral antibiotics

D. A broad spectrum oral antibiotic

21. The correct rate for ventilating a newborn is:

A. 20 breaths per minute

B. 30 breaths per minute

C. 40 breaths per minute

D. 80 breaths per minute

22. To help prevent heat loss, the newborn should be

A. Dried thoroughly immediately after birth

B. Dried thoroughly after the cord has been cut

C. Dried thoroughly and covered with a clean cloth immediately after birth

D. Covered with a clean, dry cloth after the cord has been cut

23. Newborn cord care involves

A. Applying a dry dressing to the cord stump

B. Swabbing the cord stump with alcohol and applying a dry dressing

C. Keep the cord stump dry without putting any substance on it

D. Covered with antiseptic soaked wet gauze

24. A pregnant woman who is experiencing convulsions should be:

A. Physically restrained to keep her from injuring herself.

B. Placed flat on her back.

C. Left alone in a quiet room.

D. Protected from objects that may injure her.

25. The steps in active management of the third stage of labour should be performed in the following order:

A. 1) Controlled cord traction with counter-traction to the uterus to deliver the placenta, 2) uterine massageafter the placenta is delivered, and 3) IM administration of Oxytocin.

B. 1) IV administration of Oxytocin, 2) cord clamping and cutting, and (3) uterine massage after the placentais delivered.

C. 1) Cord clamping and cutting, 2) controlled cord traction, and (3) IM Ergometrine administration.

D. 1) Intramuscular injection of Oxytocin, 2) controlled cord traction with counter-traction to the uterus to deliver the placenta, and (3) uterine massage after placenta is delivered

26. When you offer the pregnant woman HIV testing services, you should:

1. Advise her and allow her to decide whether or not to take the HIV test.
2. Advise her to obtain permission from her husband before deciding to take the HIV test.
3. Perform the HIV test without informing the patient.
4. Tell the patient that she should take the HIV test for the baby’s benefit

27. After each vaginal exam, the following information should be recorded on the Parthograph:

A. Cervical dilation.

B. Fetal heart rate.

C. Descent of the fetal head.

D. Molding of the fetal skull.

E. A and C are correct.

F. A and D are correct.

28. The vital signals include the following:

A. Blood type, blood pressure, pulse, and urinalysis.

B. Pulse, blood pressure, blood type, and respiration.

C. Temperature, fetal heart rate, contractions, and blood pressure.

D. Pulse, respiration, temperature, and blood pressure.

29. When advising the mother about breastfeeding, a competent health care provider should tell her to:

A. Avoid giving the newborn too much colostrum.

B. Establish a breastfeeding schedule, this way the baby has time to sleep.

C. Give the baby water after breastfeeding.

D. Breastfeed as often as the baby demands and for as long as he/she wishes to feed.

30. A puerperal infection can be diagnosed when:

A. The woman claims tiredness

B. There is an increase in the woman’s temperature (>38 ºC) and pulse rate

C. The woman complains of being hot.

D. There is no increase in temperature, but the pulse rate increases.

31. You have just attended a normal birth. Before removing your gloves, what should you do with the contaminated instruments used during the birth?

A. Place them in a bucket so that the supportive staff may wash them with water and soap
B. Submerge them in a 0.5% chloride solution for 10 minutes.

C. Submerge them in a 0.5% chloride solution for 30 minutes.

D. Wash the instruments with water and soap, then place them in a 0.5% chloride solution.

**Part II- True/ False Questions**

1. Rapid initial assessment should be carried out on all women of childbearing age who present with a problem.
2. Continuous slow bleeding or sudden bleeding after childbirth requires early and aggressive intervention.
3. Active management of the third stage of labor should be practiced only on women who have a history of postpartum hemorrhage.
4. If a retained placenta is undelivered after 30 minutes of oxytocin administration and the uterus is contracted, controlled cord traction and fundal pressure should be attempted.
5. The presenting signs and symptoms of eclampsia include convulsions, diastolic blood pressure of 90 mm Hg or more after 20 weeks gestation and proteinuria of 2+ or more.
6. The drug of choice for preventing and treating convulsions in severe preeclampsia and eclampsia is diazepam.
7. Cervical dilation plotted to the right of the alert line on the Parthograph indicates unsatisfactory progress of labor.
8. Lower abdominal pain and uterine tenderness, together with foul-smelling lochia, are characteristic of metritis.
9. When using a bag and mask to resuscitate a newborn, the newborn‘s neck must be slightly extended to open the airway.
