# Supplementary material for: Quality of and barriers to routine childbirth care signal functions in primary level facilities of Tigray, Northern Ethiopia: Mixed method study
Source: PLoS One. 2020 Jun 12;15(6):e0234318. doi: 10.1371/journal.pone.0234318 (PMC7292403; doi:10.1371/journal.pone.0234318)
Supplement: Attachment — Submitted filename: Review report_PLOS One D- 19-31921.docx [file pone.0234318.s006.docx]

Review report of PLOS One: D- 19-31921

Title of the paper: Quality and Barriers to Routine Childbirth Care Signal Functions in Primary Level Facilites of Tigray, Northern Ethiopia: Mixed Method Study

| Summary | Overall the paper is good. The authors have successfully addressed the main objective of the paper. They have applied different data collection techniques and triangulate those very appropriately. Ethical procedure was followed rightly for protocol and obtaining written consent during data collection. It was easy to follow as the introduction, method, result and conclusion section were well organized and supported every section.  However, some of the section particularly in the methodology section should be clearer. |
| --- | --- |
| Major issues | In the methodology section, overall line numbers 118-173 for qualitative part should rewrite; for example, data collection methods, participants category and data organization .  Specifically, line numbers 118-119, 155-158, please check the sentences and make consistence when you write about the study participants. In the first sentence you have written about applying qualitative approach among Skilled Birth Attendant and in the sentences lines 155-158 you have mentioned about 12 key informants. So, there has two types of participants and you have used two type data collection tools for qualitative data collection, but you have written separately which is confusing.  In the line numbers 229-233 Please write more detail about qualitative data preparation, coding, theme selection and content analysis. Did you use any software for data coding or analysis? If so, please mention. |
| Minor issues | i.In the abstract; line number 30, you have mentioned focus group , it’s actually Focus Group Discussion (FGD) a specific method name. Please write Focus Group Discussion.  ii. Line no 31-32 , you have used validated tools. Would you please explain how the tools were validated. If it is through field test prior to use for filed data collection you should mention it here.  iii. Did you prepare any verbatim transcript for interpretation. Verbatim interpretion words are not clear. Line 34-35  iv. The Abstract needs some modification, in result section while stating the predictors of quality of care, in bracket including the p values along with coefficient value and confidence intervals would portray the predictors more clearly [line 37 to line 42].  v. Line 49-59 conclusion section can be a bit shorter focusing the main points in few sentences.  vi. Line 79 Please use capital letter before the writer name, Donabedian  vii. Line 84 please write quality of care instead of only quality  viii. Line 121-122, 49.7% population are male? Why did you use this data, not clear.  ix. Title: Line number 275, as you have described barrier of providers in this section. Could you please include the word into your title? It might give idea about the section to the reader when they will see the title of the paragraph.  x.You have given reference for some quotation; for instance Line no 300. Reference interview 6 . Instead of interview 6, can you please write the interview of skilled birth attendants or a key informant interview.  xi. In Result section, while interpreting Table 6 i.e. Barriers for Quality of Routine Childbirth Care Signal Functions, interpretation could be written differently, instead of stating the results in same way, for some variables you could interpret as, due to change in independent variable the predicted variable increased significantly or otherwise [line 320 to line 337]. It is not mandatory to mention the adjusted coefficient value each time. Moreover, while you are stating the 95% confidence interval and p-value, you should also include the beta value i.e. co-efficient value. I think this section needs some tightening.  xii.Line number 370 write mixed method approach  xiii. In line number 373 you can write over patient flow for delivery instead of delivery load. |
| Other comments | Please check formatting and some spelling while you go through the paper. |
