# Supplementary material for: Quality of and barriers to routine childbirth care signal functions in primary level facilities of Tigray, Northern Ethiopia: Mixed method study
Source: PLoS One. 2020 Jun 12;15(6):e0234318. doi: 10.1371/journal.pone.0234318 (PMC7292403; doi:10.1371/journal.pone.0234318)
Supplement: Attachment — Submitted filename: Response to Reviewers_April_12_2020_PMP.docx [file pone.0234318.s007.docx]

**Editor-in-Chief**

**PLOS ONE**

**Subject: Response to reviewers**

**Re:** PONE-D-19-31921 Version 1: April 10, 2020

Dear Dr. Brownie, Academic Editor and Respected Reviewers,

We are grateful for your consideration of our manuscript to publish at PLOS ONE. We thank the reviewers for their careful review and comments that have substantially improved our manuscript.

Responses to the editor’s and reviewers’ comments are provided below and corresponding edits have been made to the manuscript in track changes.

Please let us know if you have any further questions or suggestions.

Sincerely,

Haftom Gebrehiwot (PI)

Thank you for your guidance and assistance in improving our manuscript. We have revised the manuscript and addressed the key points raised during the review process.

**RESPONSE to Required Elements:**

| Ensure that your manuscript meets PLOS ONE's style requirements, including those for file naming. | These have been addressed in our reformatted submission. |
| --- | --- |
| Statistical probabilities of 0 to be corrected to a more appropriate limit p<0.0001. | Thank you for pointing this out. We have corrected in our revised results section. |
| Financial disclosure statement must read  “The funders had no role in study design, data collection and analysis, decision to publish or preparation of the manuscript”.   a. Please provide an amended Funding Statement that declares *all* the funding or sources of support received during this specific study (whether external or internal to your organization) as detailed online in our guide for authors at [http://journals.plos.org/plosone/s/submit-now](http://www.google.com/url?q=http%3A%2F%2Fjournals.plos.org%2Fplosone%2Fs%2Fsubmit-now&sa=D&sntz=1&usg=AFQjCNFeMGrMjJ9uhUwWGbg8DgPACiSGSQ). b. Please state what role the funders took in the study.  If any authors received a salary from any of your funders, please state which authors and which funder. If the funders had no role, please state: "The funders had no role in study design, data collection and analysis, decision to publish, or preparation of the manuscript."   Please include your amended statements within your cover letter; we will change the online submission form on your behalf. | Thank you - completed as suggested. |
| 4. We note that you have indicated that data from this study are available upon request. PLOS only allows data to be available upon request if there are legal or ethical restrictions on sharing data publicly. For information on unacceptable data access restrictions, please see [http://journals.plos.org/plosone/s/data-availability#loc-unacceptable-data-access-restrictions](http://www.google.com/url?q=http%3A%2F%2Fjournals.plos.org%2Fplosone%2Fs%2Fdata-availability%23loc-unacceptable-data-access-restrictions&sa=D&sntz=1&usg=AFQjCNEXzPsL3-0cuRidr75Jqop1ttsrSA).  In your revised cover letter, please address the following prompts:  a) If there are ethical or legal restrictions on sharing a de-identified data set, please explain them in detail (e.g., data contain potentially identifying or sensitive patient information) and who has imposed them (e.g., an ethics committee). Please also provide contact information for a data access committee, ethics committee, or other institutional body to which data requests may be sent.  b) If there are no restrictions, please upload the minimal anonymized data set necessary to replicate your study findings as either Supporting Information files or to a stable, public repository and provide us with the relevant URLs, DOIs, or accession numbers. Please see [http://www.bmj.com/content/340/bmj.c181.long](http://www.google.com/url?q=http%3A%2F%2Fwww.bmj.com%2Fcontent%2F340%2Fbmj.c181.long&sa=D&sntz=1&usg=AFQjCNGuuYMEnXf0bgbHKHNTmy35AeELiA) for guidelines on how to de-identify and prepare clinical data for publication. For a list of acceptable repositories, please see [http://journals.plos.org/plosone/s/data-availability#loc-recommended-repositories](http://www.google.com/url?q=http%3A%2F%2Fjournals.plos.org%2Fplosone%2Fs%2Fdata-availability%23loc-recommended-repositories&sa=D&sntz=1&usg=AFQjCNHN0-M5bDpSaCHcU6ANxrOyleJg5w).  We will update your Data Availability statement on your behalf to reflect the information you provide. | Thanks for pointing this out. We have included the data availability statement in the revised cover letter. You can update it on the online submission on behalf of me. |
| Your ethics statement must appear in the methods section of your manuscript. If your ethics statement is written in any section besides the Methods, please move it to the Methods section and delete it from any other section. Please also ensure that your ethics statement is included in your manuscript, as the ethics section of your online submission will not be published alongside your manuscript. | Thank you for your information; we have included an ethical statement in the methods section. |

**Reviewers' comments:**

| Reviewer #1 |  |
| --- | --- |
| Overall the paper is good. The authors have successfully addressed the main objective of the paper. They have applied different data collection techniques and triangulate those very appropriately. Ethical procedure was followed rightly for protocol and obtaining written consent during data collection. It was easy to follow as the introduction, method, result and conclusion section were well organized and supported every section. However, some of the section; particularly in the methodology section should be clearer. | Thank you for the acknowledgements of our efforts and the positive reinforcement.  Based on your comments we have made major revisions in our methods section. Many of these revisions are reflected in the comments below. |
| Major issues:  In the methodology section, overall line numbers 118-173 for qualitative part should rewrite; for example, data collection methods, participants’ category and data organization should write consistently and detail. Specifically, line numbers 118-119, 155-158, please check the sentences and make consistence when you write about the study participants. In the first sentence you have written about applying qualitative approach among Skilled Birth Attendant and in the sentences lines 155-158 you have mentioned about 12 key informants. So, there has two types of participants and you have used two type data collection tools for qualitative data collection, but you have written separately which is confusing.  In the line numbers 229-233 Please write more detail about qualitative data preparation, coding, theme selection and content analysis. Did you use any software for data coding or analysis? If so, please mention. | Thanks for pointing this out. We have corrected and rewritten. Please look at the track change of methods section, specifically qualitative approach was written separately. Line numbers, 192-211  Yes, we have used Atlas ti^TM^ 7.5 software for data coding and analysis. We have mentioned it out. Look track change line numbers 269-286.. |
| Minor issues:  i. In the abstract; line number 30, you have mentioned focus group, it’s actually Focus Group Discussion (FGD) a specific method name. Please write Focus Group Discussion.  ii. Line no 31-32, you have used validated tools. Would you please explain how the tools were validated. If it is through field test prior to use for filed data collection you should mention it here.  iii. Did you prepare any verbatim transcript for interpretation? Verbatim interpretation words are not clear. Line number 34-35  iv. The Abstract needs some modification, in result section while stating the predictors of quality of care, in bracket including the p values along with coefficient value and confidence intervals would portray the predictors more clearly [line 37 to line 42].  v. Line 49-59 conclusion section can be a bit shorter focusing the main points in few sentences.  vi. Line 79 Please use capital letter before the writer name, Donabedian.  vii. Line 84 please write quality of care instead of only quality  viii. Line 121-122, 49.7% population are male? Why did you use this data, not clear?  ix. Title: Line number 275, as you have described barrier of providers in this section. Could you please include the word into your title? It might give idea about the section to the reader when they will see the title of the paragraph  x. You have given reference for some quotation; for instance Line no 300. Reference interview 6. Instead of interview 6, can you please write the interview of skilled birth attendants or a key informant interview?  xi. In Result section, while interpreting Table 6 i.e. Barriers for Quality of Routine Childbirth Care Signal Functions, interpretation could be written differently, instead of stating the results in same way, for some variables you could interpret as, due to change in independent variable the predicted variable increased significantly or otherwise [line 320 to line 337]. It is not mandatory to mention the adjusted coefficient value each time. Moreover, while you are stating the 95% confidence interval and p-value, you should also include the beta value i.e. co-efficient value. I think this section needs some tightening.  xii. Line number 370 write mixed method approach  xiii. In line number 373 you can write over patient flow for delivery instead of delivery load.  Other comments: In addition, please check formatting and some spelling while you go through the paper. | Thanks, we fully agreed and mentioned as you indicated.  We have used a validated tool from other preceding validated study of our selves. Actually this study is a validation study of the quality care indicators of routine childbirth care in our setting, which has been submitted to the journal of BMC-Reproductive health and it is under second review process. Almost near to be accepted. For the key information how, we can validate the tool you can read the methods section of this manuscript.  Yes, we have prepared verbatim transcripts for interpretation of the words. In the abstract section you right, however, you can access in the result section.  Agreed and concur with your feedback. We have modified it accordingly. Please check the result section under Abstract.  Thank you. We have agreed and shorten the conclusion section focusing on the main points only.  Accept and corrected.  Accept and corrected.  This is just to describe the study setting, about Tigray regional state, one of the nine proveniences in Ethiopia. About the percentage of the population, we are trying to mention the proportion of female from the total population. This implies that 50.3% of the total population of Tigray is female. But, this percentage did not show our study population. Anyway, for making it clear, we have corrected it.  Thanks for pointing this out. We have included as per your suggestion.  Accepted and corrected.  Accepted and corrected. Please check the corrected one under the result section of tracked change manuscript  Accepted and corrected.  Accepted, but still those mothers are not patients rather they are clients. That is why we corrected and replaced the word delivery load by client flow for delivery.  Thank you for your suggestion. We have improved the language throughout the manuscript, particularly the abstract and method sections. |
| Reviewer #2:  This is an interesting manuscript. However, there are some issues that need to be addressed | Thank you for your interest and suggestion |
| 1. There are a lot of grammatical errors in the manuscript and I recommend revising the entire manuscript, see examples below:   - A total of twelve interviewee were conducted (page 14, lines 157-158). - We were used principal component analysis (page 15, line 184)  - In addition, PCA was done to create an index score. Prior to perform PCA, the suitability of data was assessed (page 16, lines 219-220).   2. The authors should consider presenting the results of the qualitative data analysis separate from that of the quantitative data, and in doing do should provide additional information of the participants and not just FGD participant or Interviewee 6. For example highlighting their role/occupation. 3. In addition, when presenting the results of the qualitative interviews, the authors should separate the responses of participants, for example on page 20, the authors lumped together the responses for interviewee 6 and 8. 4. The authors should consider providing a Good Reporting of a Mixed Methods Study (GRAMMS) checklist. | Thanks for pointing this out. We have corrected and revised.  Openly speaking, the author’s main interest/priority decision was a QUANTITATIVE approach explained through a QUALITATIVE follow up. That is why we collect the data at different times. We believe that, there are many types of mixing method researches. But, here our focus is qualitative study depends on quantitative results. We didn’t mean that, it is not possible to do separate data analysis, but the researchers’ insight and nature of the problem matters. In explanatory sequential mixed method design, usually quantitative data collection and analysis is the priority, then after you can emanate research questions from the first analysis that needs further explained key findings.  About, considering additional information of the participants for example highlighting their role/occupation: Thanks accepted and corrected this one as per your suggestion.  Thank you for your suggestion. We have corrected.  We thank you again for your wonderful observation. We have tried our best level to considered the GRAMMS (reason why, design, data collection analysis, integration and limitation) while we write. |
